# An allosteric gating model recapitulates the biophysical properties of *I*_K,L_ expressed in mouse vestibular type I hair cells

**DOI:** 10.1113/JP274202

**Published:** 2017-09-24

**Authors:** Paolo Spaiardi, Elisa Tavazzani, Marco Manca, Veronica Milesi, Giancarlo Russo, Ivo Prigioni, Walter Marcotti, Jacopo Magistretti, Sergio Masetto

**Affiliations:** ^1^ Department of Brain and Behavioural Sciences University of Pavia Pavia 27100 Italy; ^2^ Instituto de Estudios Inmunológios y Fisiopatológicos (IIFP) ‐ CONICET Universidad Nacional de La Plata La Plata 1900 Argentina; ^3^ Department of Biomedical Science University of Sheffield Sheffield S10 2TN UK; ^4^ Department of Biology and Biotechnology University of Pavia Pavia 27100 Italy

**Keywords:** channel gating model, IK,L, type I vestibular hair cell

## Abstract

**Key points:**

- Vestibular type I and type II hair cells and their afferent fibres send information to the brain regarding the position and movement of the head.
- The characteristic feature of type I hair cells is the expression of a low‐voltage‐activated outward rectifying K^+^ current, *I*_K,L_, whose biophysical properties and molecular identity are still largely unknown.
- *In vitro*, the afferent nerve calyx surrounding type I hair cells causes unstable intercellular K^+^ concentrations, altering the biophysical properties of *I*_K,L_.
- We found that in the absence of the calyx, *I*_K,L_ in type I hair cells exhibited unique biophysical activation properties, which were faithfully reproduced by an allosteric channel gating scheme.
- These results form the basis for a molecular and pharmacological identification of *I*_K,L_.

**Abstract:**

Type I and type II hair cells are the sensory receptors of the mammalian vestibular epithelia. Type I hair cells are characterized by their basolateral membrane being enveloped in a single large afferent nerve terminal, named the calyx, and by the expression of a low‐voltage‐activated outward rectifying K^+^ current, *I*_K,L_. The biophysical properties and molecular profile of *I*_K,L_ are still largely unknown. By using the patch‐clamp whole‐cell technique, we examined the voltage‐ and time‐dependent properties of *I*_K,L_ in type I hair cells of the mouse semicircular canal. We found that the biophysical properties of *I*_K,L_ were affected by an unstable K^+^ equilibrium potential (*V*_eq_K^+^). Both the outward and inward K^+^ currents shifted *V*_eq_K^+^ consistent with K^+^ accumulation or depletion, respectively, in the extracellular space, which we attributed to a residual calyx attached to the basolateral membrane of the hair cells. We therefore optimized the hair cell dissociation protocol in order to isolate mature type I hair cells without their calyx. In these cells, the uncontaminated *I*_K,L_ showed a half‐activation at –79.6 mV and a steep voltage dependence (2.8 mV). *I*_K,L_ also showed complex activation and deactivation kinetics, which we faithfully reproduced by an allosteric channel gating scheme where the channel is able to open from all (five) closed states. The ‘early’ open states substantially contribute to *I*_K,L_ activation at negative voltages. This study provides the first complete description of the ‘native’ biophysical properties of *I*_K,L_ in adult mouse vestibular type I hair cells.

## Key points

- Vestibular type I and type II hair cells and their afferent fibres send information to the brain regarding the position and movement of the head.
- The characteristic feature of type I hair cells is the expression of a low‐voltage‐activated outward rectifying K^+^ current, *I*_K,L_, whose biophysical properties and molecular identity are still largely unknown.
- *In vitro*, the afferent nerve calyx surrounding type I hair cells causes unstable intercellular K^+^ concentrations, altering the biophysical properties of *I*_K,L_.
- We found that in the absence of the calyx, *I*_K,L_ in type I hair cells exhibited unique biophysical activation properties, which were faithfully reproduced by an allosteric channel gating scheme.
- These results form the basis for a molecular and pharmacological identification of *I*_K,L_.

**Abbreviations**
erg: ether‐a‐go‐go
G(norm): normalized conductance
HH: Hodgkin‐Huxley
*n*: number of cells
P: postnatal day
*R*_s_: series resistance
RT: room temperature
SCCs: semicircular canals with the ampulla
τ_f_: fast time constant
τ_s_: slow time constant
VC: voltage‐clamp
*V*_cond_: conditioning potential
*V*_eq_K^+^: equilibrium potential for K^+^
*V*_hold_: holding potential
*V*_rev_K^+^: K^+^ reversal potential
$V_{R_{s}}$: voltage drop over residual *R*_s_
*V*_test_: test potential

## Introduction

Vestibular hair cells are responsible for relaying information about head movements to the central nervous system via afferent vestibular nerve fibres. Incoming stimuli are transduced by the mechano‐gated ion channels positioned at the top of stereocilia, which project from the apical surface of the hair cells. The opening of the transducer channels causes hair cells to depolarize and the release of the neurotransmitter glutamate onto the afferent fibres mediated by the activation of voltage‐dependent L‐type 1.3 Ca^2+^ channels (Almanza *et al*. 2003; Bao *et al*. 2003; Bonsacquet *et al*. 2006; Zampini *et al*. 2006). In higher vertebrates, vestibular peripheral processing employs two distinct sensory cells: type I hair cells, which are innervated by a single afferent nerve calyx enveloping their basolateral membrane, and type II hair cells, which are contacted by 10–20 afferent bouton nerve terminals (Songer & Eatock 2013). The physiological interaction between type I hair cells and the afferent nerve calyx is still unclear, although a recent study has provided evidence that this interaction preserves the fidelity of high speed synaptic transmission (Contini *et al*. 2016). One of the most distinctive characteristic of type I hair cells is the expression of the negatively activated outward K^+^ current *I*_K,L_, such that it is almost fully available at the cell's resting membrane potential (Rennie & Correia 1994; Rüsch & Eatock 1996; Rüsch *et al*. 1998; Hurley *et al*. 2006). The molecular nature of *I*_K,L_ is still unclear (Meredith & Rennie 2016), although both KCNQ and ether‐a‐go‐go (erg) K^+^ channel subunits have been suggested to contribute (Hurley *et al*. 2006). A major problem in defining the biophysical properties of *I*_K,L_ is that they seem to vary not only from cell to cell but also during postnatal development and within the same type I hair cell over the recording time (Ricci *et al*. 1996; Rüsch & Eatock 1996; Hurley *et al*. 2006; Contini *et al*. 2012).

Recently, it has been shown that *I*_K,L_ activation causes the accumulation of K^+^ in the synaptic cleft between the basolateral membrane of type I hair cells and the residual calyx, which leads to a depolarizing shift of the K^+^ current reversal potential (*V*_rev_K^+^) (Lim *et al*. 2011; Contini *et al*. 2012, 2016). We tested the hypothesis that the biophysical properties of *I*_K,L_ are largely dependent on the intercellular K^+^ accumulation, by performing different experiments aimed at minimizing the effect of the residual calyx, and associated intercellular K^+^ accumulation. Recordings were obtained from type I hair cells *in situ* or dissociated from semicircular canal cristae. We found that the ‘native’ kinetic properties of *I*_K,L_ investigated in the absence of the calyx were similar between cells and during long‐lasting recordings, though some variability in *I*_K,L_ activation range was present. *I*_K,L_ exhibited a complex activation and deactivation time course, with multiple kinetic components, which could be modelled by a single channel allosteric gating model where the open state can be reached by five, intercommunicating closed states.

## Methods

### Ethical approval

All procedures used were approved by the Ministero Italiano della Salute (Rome, Italy) and animal experiments were carried out in accordance with the European Communities Council Directive of 24 November 1986 (86/609/EEC).

### Whole crista preparation

Mice (Swiss CD1) from both sexes were obtained from Charles River (Italy). Following brief anaesthesia via inhalation with 2‐bromo‐2‐chloro‐1,1,1‐trifluoroethane (99%, Sigma‐Aldrich), mice were decapitated and semicircular canals with their ampullae (SCCs) were surgically removed in chilled extracellular solution (Extra_std, in mm): NaCl 135, CaCl_2_ 1.3, KCl 5.8, MgCl_2_ 0.9, Hepes 10, glucose 5.6, NaH_2_PO_4_ 0.7, sodium pyruvate 2. Vitamins (Gibco Invitrogen, Grand Island, NY, USA, 10 mL L^−1^) and amino acids (Gibco Invitrogen, 20 mL L^−1^) were also added. The pH was adjusted to 7.4 with NaOH (final osmolality: 310 mOsm kg^−1^).

After dissection of the SCC, the roof of the ampulla was gently torn to expose the crista ampullaris and the preparation was then immobilized at the bottom of the recording chamber by mean of a weighted nylon mesh. Hair cells in the crista were viewed by using an upright microscope equipped with differential interference contrast optics (Olympus, Japan) and a 60× water immersion objective. In order to reach the basolateral membrane of the hair cells positioned within the whole crista preparation, a ‘cleaning’ pipette was used to remove the tissue above the targeted cell (see Contini *et al*. 2012 for further details). The bath solution (Extra_std) was continuously changed (0.5 ml min^−1^) by mean of a peristaltic pump (Gilson, France). *In situ* recordings were obtained from 23 type I hair cells from mice ranging from postnatal day 6 (P6) to P19.

### Hair cell dissociation

For hair cell dissociation, the SCCs were subjected to different enzymatic treatments. The isolated SCCs were transferred to a Petri dish containing the dissociation extracellular solution (Extra_D, in mm): NaCl 138, CaCl_2_ 0.1, KCl 5.8, MgCl_2_ 0.9, Hepes 10, glucose 5.6, NaH_2_PO_4_ 0.7, sodium pyruvate 2, vitamins (10 mL L^−1^) and amino acids (20 mL L^−1^); pH 7.4, osmolality 310 mOsm kg^−1^. Crude papain (Calbiochem‐Nova Biochem Corporation, USA; 0.9 mg ml^−1^) and l‐cysteine (Sigma Aldrich; 0.3 mg ml^−1^) were added to the above solutions and SCCs were incubated for 40 min at 37°C. Then, SCCs were transferred to a Petri dish containing Extra_D plus bovine serum albumin (Sigma Aldrich; 1 mg ml^−1^) for 40 min at room temperature (RT, 22–25°C) to stop the enzymatic activity. Then, the ampullae were transferred onto the recording chamber filled with the recording extracellular solution (mm): NaCl 135, CaCl_2_ 1.3, KCl 5.8, MgCl_2_ 0.9, Hepes 10, glucose 5.6, NaH_2_PO_4_ 0.7, sodium pyruvate 5, plus vitamins (Gibco Invitrogen, 10 mL L^−1^) and amino acids (Gibco Invitrogen, 20 mL L^−1^); pH 7.4 with NaOH, for a final osmolality of ∼314 mOsm kg^−1^. Each crista ampullaris was brushed with an eyelash and smeared onto the glass‐bottom of the recording chamber to dislodge the hair cells from the epithelium. Cells were left to adhere to the bottom of the chamber for 10–15 min before recording. Recordings were obtained from 81 type I hair cells dissociated from mice ranging from P7 to P77.

### Patch‐clamp recordings

Whole‐cell recordings were obtained in voltage‐clamp (VC) mode at room temperature. The patch‐clamp amplifier was an Axopatch 200B (Axon Instruments, USA). Soda glass pipettes (Hilgenberg, Germany) were pulled to tip diameters of about 2 μm, fire‐polished and partially coated with Sylgard (Dow Corning 184, Midland, MI, USA). The micropipettes were filled with a K^+^‐based intracellular solution (in mm): KCl 131, MgCl_2_ 3, disodium phosphocreatine 10, Na_2_ATP 5, Hepes 5, EGTA 1, pH 7.2 with KOH, for a final osmolality of 293 mOsm kg^−1^. When filled with the intra‐pipette solution, micropipettes had a resistance in the bath of 2–5 MΩ. All voltages were corrected for the liquid junction potential between the intra‐pipette and the extracellular bath solution of –4 mV, which was calculated using pClamp software Junction Potential tool (version 9 or 10, Molecular Devices, USA). In order to seal the patch electrode to the basolateral membrane of hair cells, at least some of the calyx had to be removed, which was done mechanically by the patch pipette. Another patch pipette was then used for the recording. The pipette resistance was kept as low as possible, despite the greater difficulty in obtaining a gigaseal, to minimize the series resistance (*R*_s_). A low pipette resistance and a good ‘rupturing’ of the membrane patch gave acceptably low *R*_s_ (<9 MΩ; for larger *R*_s_ values, experiments were discarded). *R*_s_ and the cell membrane capacitance were calculated off‐line by the capacitative artifact elicited by a voltage step from –124 mV, at which *I*_K,L_ was fully deactivated, to –44 mV, where *I*_K,L_ activated slowly enough to appreciate the whole artifact. When the voltage drop across the residual *R*_s_ ($V_{R_{s}}$) produced by inward or outward currents was >6 mV the recordings were discarded. Voltages were not corrected for $V_{R_{s}}$ except for those used for the activation curve of *I*_K,L_.

The amplifier's filter bandwidth was set at 10 kHz. Digital sampling frequency was five times the analog bandwidth of the signal recorded. Current and voltage were measured and controlled through a DigiData 1322A or 1440 interface (AD/DA converter; Molecular Devices, USA) connected to a computer running pClamp software.

### Data analysis

Analysis of traces and results were performed with Clampfit (pClamp version 10, Molecular Devices), Origin 6.1 (OriginLab, USA) and Microsoft Excel (Microsoft Corporation, USA).

*I*_K,L_ steady‐state activation curve was generated by delivering multiple voltage (conditioning) steps from a holding potential of –64 mV and measuring instantaneous tail current amplitude at the test potential of –44 mV. Sigmoidal curves were fitted with the following Boltzmann function:

(1) I(V)=I max +(I min −I max )/(1+e(V−V1/2)/S),

where *I(V)* is current at voltage *V*, *I*_min_ and *I*_max_ are minimum and maximum currents, *V*_1/2_ is voltage corresponding to half‐maximal activation, and *S* is the voltage corresponding to an e‐fold increase in *I(V)*.

### Markov model

The macroscopic *I*_K,L_ (in arbitrary units, a.u.) was simulated by multiplying the normalized maximal conductance for the fraction of K^+^ channels in the open states, which would be the equivalent to the probability of a single *I*_K,L_ channel to occupy those states. The reversal potential for *I*_K,L_ was estimated to be –76 mV. *I*_K,L_ channel open and closed times were obtained by numerical resolution of the gating scheme shown in Fig. 6 for time and voltage. The probability of occupancy of each state as a function of time for the corresponding Markov model was obtained by Euler integration of the master equation taking a step size of 0.1 ms. We checked that the latter step size was small enough by verifying that the results are independent of the step size chosen. Kinetic parameters, which are reported in the legend of Fig. 6, were iteratively adjusted to satisfactory reproduce the mean *I*_K,L_ kinetics obtained experimentally.

### Statistics

Data are expressed as the means ± standard deviation (SD), unless otherwise stated; *n* = number of cells.

## Results

### The residual calyx precludes characterization of the true properties of *I*_K,L_

Mouse type I hair cells express two outward rectifying K^+^ currents: *I*_K,v_, which activates close to –50 mV, and *I*_K,L_, which activates close to –90 mV and is fully activated at –60 mV (Rennie & Correia 1994; Rüsch & Eatock 1996; Rüsch *et al*. 1998; Li *et al*. 2010; Contini *et al*. 2012). Therefore, some of the kinetic properties of *I*_K,L_ can be studied in isolation by exploiting the different voltage range of activation.

**Figure 1:**
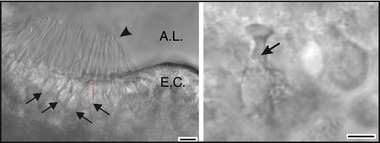
Crista preparation Left panel, photomicrograph of a portion of the crista preparation (P7). A few hair cells are indicated by arrows (the basolateral profile of one is also shown by a dashed red line). The hair bundles protruding from the sensory epithelium into the ampullary lumen (A.L.) are indicated by the arrowhead. Type I hair cells were visually identified by their amphora‐like shape. The eminentia cruciata (E.C.) is the region of the vertical cristae devoid of hair cells. Scale bar, 10 μm. Right panel, representative photomicrograph of a type I hair cell before recording (P17). Scale bar, 5 μm. The arrow points at the border between the residual calyx and the ‘naked’ portion of the type I cell, where the patch pipette tip was sealed.

In a first set of experiments, recordings were obtained from mouse type I hair cells maintained in an *in situ* preparation (Fig. 1, left panel). A residual nerve calyx enveloping at least part of the basolateral region of the investigated cell was sometimes obvious (arrow in Fig. 1, right panel). However, in most experiments the residual calyx was not visually detectable, although it was deducible from alterations in the electrophysiological recordings, as described below.

**Figure 2:**
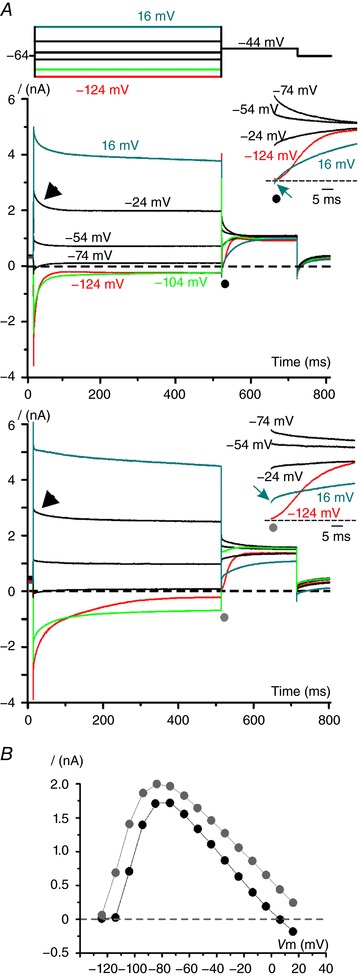
Patch‐clamp recordings from *in situ* crista hair cells *A*, representative voltage‐clamp (VC) responses obtained from a type I hair cell (P14) at the beginning (upper panel) and 4 min after the start of the recordings (lower panel). Currents were elicited by hyperpolarizing and depolarizing conditioning voltage steps (*V*_cond_: 10 mV increments) from the holding potential of –64 mV, shown schematically above the upper panel. The tail currents were measured at –44 mV after the *V*_cond_ steps. The horizontal dashed line, in this and the following figures, represents the zero‐current level. The red, green and blue traces highlight responses to *V*_cond_ steps of –124 mV, –104 mV and 16 mV, respectively. Outward K^+^ currents show fast partial relaxation (arrowhead). The insets show the first portion of the tail currents on an expanded scale. Note that, in the selected group of traces, the amplitude of the outward instantaneous tail current was maximal following *V*_cond_ of –74 mV, and then decreased with *V*_cond_ depolarization and inverted for *V*_cond_ positive to 16 mV. *I*_K,L_ activation time course at –44 mV can be appreciated following *V*_cond_ of –124 mV (red trace), consistent with complete *I*_K,L_ deactivation at this membrane voltage. *B*, instantaneous tail current amplitude at –44 mV as a function of *V*_cond_. Filled black circles refer to tail current amplitudes measured at the beginning of the experiment, filled grey circles after 4 min. Same symbols are shown also in *A* (also in insets), indicating the time point at which the (instantaneous) tail current amplitude was measured.

Representative macroscopic currents from a type I hair cell recorded soon after achieving the whole‐cell configuration are shown in Fig. 2 *A* (upper panel). Since *I*_K,L_ is already fully activated at –60 mV, hyperpolarizing or depolarizing conditioning voltage (*V*_cond_) steps from the holding potential (*V*_hold_) of –64 mV produced large instantaneous inward or outward K^+^ currents flowing through *I*_K,L_ channels. *I*_K,L_ then deactivated during hyperpolarized *V*_cond_ steps or, for depolarized *V*_cond_ steps, it increased in size because of the larger driving force for K^+^. At voltages above –50 mV, the delayed rectifier K^+^ current *I*_K,v_ was also recruited.

Tail currents were measured in response to a fixed voltage step (*V*_test_) of –44 mV following different *V*_cond_ steps. The *I*_K,L_ activation time course was fully appreciable at *V*_test_ following a *V*_cond_ of –124 mV, at which voltage *I*_K,L_ is completely deactivated (Fig. 2 *A*, red trace). The first anomaly in the above recordings is the initial pronounced relaxation of the outward current elicited by depolarized *V*_cond_ steps, since *I*_K,L_ does not inactivate. A second and more striking abnormal feature is that, for *V*_cond_ steps less negative than –100 mV, the outward instantaneous tail current (Fig. 2 *A*, filled black circles) increased up to *V*_cond_ of –74 mV, but then it decreased with further *V*_cond_ depolarization and reversed from *V*_cond_ positive to about 16 mV (blue arrow in the inset, the instantaneous current is inward). Based on the intracellular and extracellular solutions, the estimated Nernst equilibrium potential for K^+^ (*V*_eq_K^+^) is –80 mV. Therefore, the instantaneous inward current indicates a significant (about 40 mV) rightward shift in the K^+^ current reversal potential (*V*_rev_K^+^), most likely due to K^+^ accumulation in the synaptic cleft in between the cells basolateral membrane and the residual calyx (see also Lim *et al*. 2011; Contini *et al*. 2012). This K^+^ accumulation is rapidly produced by K^+^ exiting the hair cell during depolarized *V*_cond_ steps. Likewise, hair cell repolarization, by decreasing the outward K^+^ current, reduces K^+^ accumulation. Note that the tail current at –44 mV, following *V*_cond_ of 16 mV (blue trace), was initially inward but then it reversed to outward within a few milliseconds (Fig. 2 *A*, inset) and its size increased to a steady level consistent with a more negative *V*_rev_K^+^ (i.e. closer to the theoretical value of –80 mV). These findings suggest that, because of the residual calyx, *V*_rev_K^+^ varies depending on the amount of K^+^ exiting the hair cell, which precludes the characterization of *I*_K,L_ voltage‐dependent properties. This is also evident from the curves of the instantaneous tail current amplitude as a function of *V*_cond_ (Fig. 2 *B*, filled black circles). Instead of the expected sigmoidal activation, consistent with the progressive activation of *I*_K,L_, the outward current reached a peak at –84 mV, and then decreased and even reversed at –24 mV.

In most cells showing intercellular K^+^ accumulation the properties of the macroscopic current changed rapidly during the recording, which we attributed to the deterioration of the calyx over time. Figure 2 *A* (lower panel) shows the macroscopic currents recorded from the same cell as in Fig. 2 *A* (upper panel) but 4 min later. The increased amplitude of the outward current was associated with a minor current relaxation (compare arrowheads in the upper and lower panels). Moreover, the instantaneous tail current at –44 mV, following *V*_cond_ at 16 mV, was no longer inward (compare blue traces). The above evidence suggests that over time the same voltage protocol produces less intercellular K^+^ accumulation when compared to that at the beginning of the experiment. This is also obvious from the current–voltage relation shown in Fig. 2 *B* (filled grey circles). These results can be explained by assuming that, during the experiment, a progressive structural deterioration of the damaged calyx allows a better exchange of the intercellular space with the bath solution. Similar results were also obtained in 23 additional type I hair cells. We also noticed that the deactivation kinetics at the most hyperpolarizing steps became significantly slower over the recording time (compare red traces in Fig. 2 *A* upper and lower panels), which can also be explained by the calyx deterioration. At the beginning of the experiment, negative *V*_cond_ steps eliciting inward K^+^ currents will produce a significant leftward shift in *V*_rev_K^+^ as a consequence of intercellular K^+^ depletion. This will produce an apparent speed‐up of *I*_K,L_ deactivation time course since the inward K^+^ current will appear to decrease both for *I*_K,L_ deactivation and for the concomitant leftward shift of *V*_rev_K^+^.With calyx deterioration, the contribution of the latter event will decrease, resulting in a slower *I*_K,L_ deactivation time course.

The above results indicate that the voltage‐ and time‐dependent properties of *I*_K,L_ cannot be reliably characterized when a residual calyx is present.

### *I*_K,L_ properties in type I hair cells dissociated from adult mice

**Figure 3:**
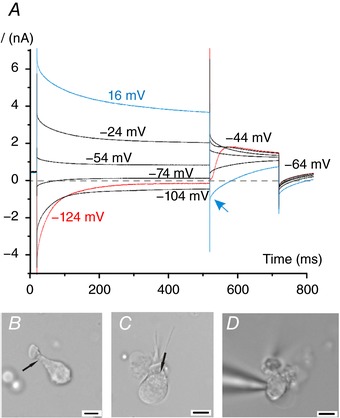
Patch‐clamp recording from a dissociated mouse crista hair cell *A*, representative ionic currents recorded from a dissociated P21 type I hair cell shown in panel *D* (the patch‐pipette tip is also visible on the left). Voltage protocol is as described in Fig. 2 *A*. Note the large relaxation of the outward currents and the instantaneous inward tail current (arrow). *B*–*D*, representative photomicrographs of dissociated hair cells. Scale bars: 5 μm.

**Figure 4:**
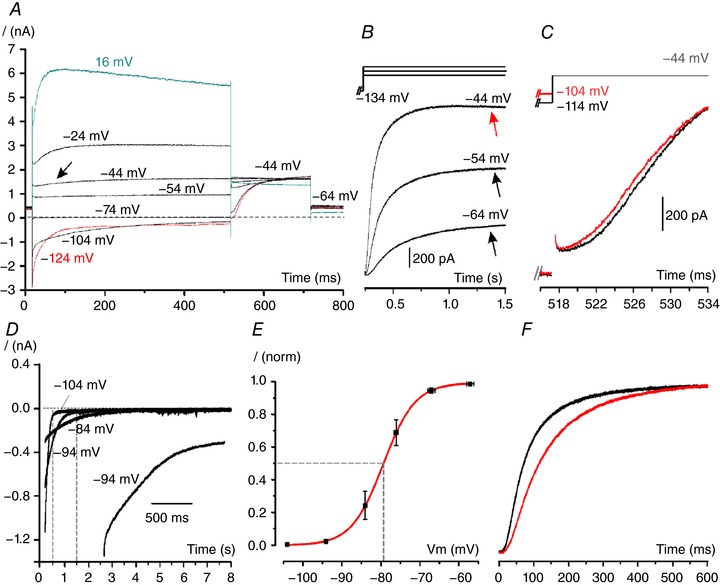
*I*_K,L_ recorded from dissociated mouse crista hair cells with no residual calyx *A*, current response recorded from a dissociated mouse type I cell (P10) showing no sign of a residual calyx (same VC protocol as in Fig. 2 *A*). Note the activation time course of a second outward rectifying K^+^ current component (*I*_K,v_) from –44 mV (arrow), and the small outward current relaxation, which was only obvious at most depolarized voltages. Also note that the instantaneous tail current amplitude in response to depolarized *V*_cond_ does not change significantly, indicating that *V*_rev_K^+^ does not vary during *V*_cond_. The residual artifact was partially blanked. *B*, representative current elicited by stepping the membrane potential of a P14 type I cell to the voltages shown above each trace, following *V*_cond_ of –134 mV to fully deactivate *I*_K,L_. Black arrows indicate a slower small outward current component at –64 mV and –54 mV. Red arrow indicates partial inactivation due to *I*_K,v_. *C*, outward current elicited in a dissociated P19 mouse type I cell at –44 mV, following a *V*_cond_ of –114 mV or –104 mV for 1 s. The delay for *I*_K,L_ activation increases by increasing conditioning hyperpolarization. *D*, representative deactivating tail currents from a P21 type I hair cell elicited by repolarizing voltage steps indicated next to the traces from the holding potential of –64 mV. Dashed vertical lines show the level of *I*_K,L_ deactivation at 0.5 s and 1.5 s. The inset shows the first 1.5 s of the same trace at –94 mV at a larger scale time. Note that the deactivation time course cannot be described by a mono‐exponential function. The residual *R*_s_ was 3 MΩ. *E*, normalized (*I*_norm_) mean *I*_K,L_ activation curve obtained by fitting the experimental data from nine cells from ≥P18 mice with a Boltzmann function (eqn (1)). Voltages were corrected for the voltage drop produced by the instantaneous tail current over the residual *R*_s_. Horizontal bars indicate ± SEM for *V*_cond_; vertical bars indicate ± SEM for *I*_norm_. *F*, normalized outward current evoked by voltage stepping at –64 mV from –134 mV in a cell with a significant *I*_K,L_ at –94 mV (black trace; P35) and in a cell with no detectable *I*_K,L_ at –94 mV (red trace; P21). *I*_K,L_ activation time course was slightly slower in the cell with the positively shifted activation curve.

In order to remove the contribution of the calyx, we tried different combinations of mechanical–enzymatic cell dissociation protocols (see Methods). Despite the enzymatic treatment, we also found evidence for substantial intercellular K^+^ accumulation in most dissociated cells. Figure 3 *A* shows a current response recorded from a dissociated type I hair cell with strikingly large intercellular K^+^ accumulation. Visually, the presence of a residual calyx enveloping the hair cell was sometimes obvious as a slight discontinuity at the calyx collar region (Fig. 3 *B* and *C*, see arrows). However, the presence of a residual calyx was not always easy to judge (Fig. 3 *D*). The survival of the calyx during the dissociation procedure is not surprising, as recordings from the calyx are mainly from dissociated hair cells (Hurley *et al*. 2006; Rennie & Streeter, 2006; Dhawan *et al*. 2010; Meredith et al. 2011, 2012; Meredith & Rennie, 2015). Nonetheless, in about 16% of dissociated type I hair cells (13 out of 81) we recorded a large outward K^+^ current (5.78 nA ± 2.25, calculated at *V*_cond_ of 16 mV; *n* = 13) which only showed a small shift in *V*_rev_K^+^ for large and long‐lasting (>1 s) depolarizing steps. These cells presumably retained none or only a small portion of the residual calyx, and as such they were considered for analysis in a limited voltage range, which did not elicit any shift in *V*_rev_K^+^. Under these recording conditions, *I*_K,L_ showed negligible relaxation, tail currents amplitude did not decrease with increasing conditioning depolarization, and *I*_K,v_ was also detectable starting from around –44 mV (Fig. 4 *A*, see arrow). It is possible that *I*_K,L_ relaxation masked the *I*_K,v_ activation time course in cells retaining a residual calyx (e.g. Figs 2 *A* and 3 *A*).

Since *I*_K,L_ properties recorded from type I hair cells of the rat utricle were shown to change during the first 2 weeks of postnatal development (Hurley *et al*. 2006), the following analysis has been performed only on mice ≥ P18. Moreover, only type I hair cells with no evidence of *I*_h_, an inward rectifying cationic current found in some type I hair cells (Rüsch & Eatock, 1996), were chosen. *I*_h_ is likely to contaminate *I*_K,L_ voltage‐ and time‐dependent properties at voltages more negative than –64 mV. In type I hair cells with no evidence of intercellular K^+^ accumulation *I*_K,L_, after an initial lag, activated with a fast sigmoidal time course followed by a slowly increasing component (arrows in Fig. 4 *B*). Outward currents were elicited at –64 mV, –54 mV and –44 mV from a hyperpolarized *V*_cond_ of –134 mV since at this potential *I*_K,v_ is fully deactivated and therefore does not contaminate *I*_K,L_ activation kinetics. A slight inactivation of the K^+^ current was present at –44 mV (red arrow), which was likely to be due to a small contribution by *I*_K,v_. We also found that the initial lag in *I*_K,L_ activation increased with *V*_cond_ hyperpolarization (Fig. 4 *C*), consistent with a Cole‐Moore shift effect (Cole & Moore, 1960) that is indicative of multiple closed channel gating states. *I*_K,L_ deactivation time course was very slow, e.g. taking several seconds to reach a steady‐state at –84 mV (Fig. 4 *D*), and could not be fitted by a single exponential function. These complex deactivation kinetics are consistent with the *I*_K,L_ channel gating model proposed below.

To build the *I*_K,L_ activation curve, hair cells were held for 8 s at *V*_cond_ steps between –104 mV and –54 mV (in 10 mV increment) followed by a voltage step to –44 mV. *I*_K,v_ is not expected to contaminate the instantaneous tail currents since at –44 mV it activates slowly. Figure 4 *E* shows the mean *I*_K,L_ activation curve obtained by fitting the data obtained from nine cells with eqn (1) (red line). The mean *V*_1/2_ was –79.65 mV (±4.35) and the mean *S* was 2.84 (± 0.41). Similar to previous studies (Hurley *et al*. 2006) we found that the *V*_1/2_ of *I*_K,L_ activation was rather variable among cells (between –73.00 and –86.53 mV), but *I*_K,L_ activation kinetics was faster in cells expressing a more hyperpolarized *I*_K,L_ activation curve (Fig. 4 *F*). The latter finding, together with the steep voltage dependence (small *S* value) of *I*_K,L_ activation curve, is consistent with type I hair cells expressing a homogenous population of ion channels whose midpoint of activation can shift along the voltage axis. However, the presence of multiple kinetics components in the activation and deactivation time course (Fig. 4 *B*–*D*) may indicate that more than one ion channel population contribute to the macroscopic current. In the classic Hodgkin‐Huxley (HH) gating model of ion channels (Hodgkin & Huxley, 1952), the activation of a current resulting from a homogenous population of ion channels is described by an exponential raised to a power, while deactivation is described by a single exponential. Additional experiments were therefore performed from a *V*_hold_ of –134 mV to investigate *I*_K,L_ activation kinetics at voltages more negative than –64 mV.

### A multi‐open‐state kinetic model of *I*_K,L_ channel gating is able to faithfully reproduce the activation and deactivation properties of *I*_K,L_

**Figure 5:**
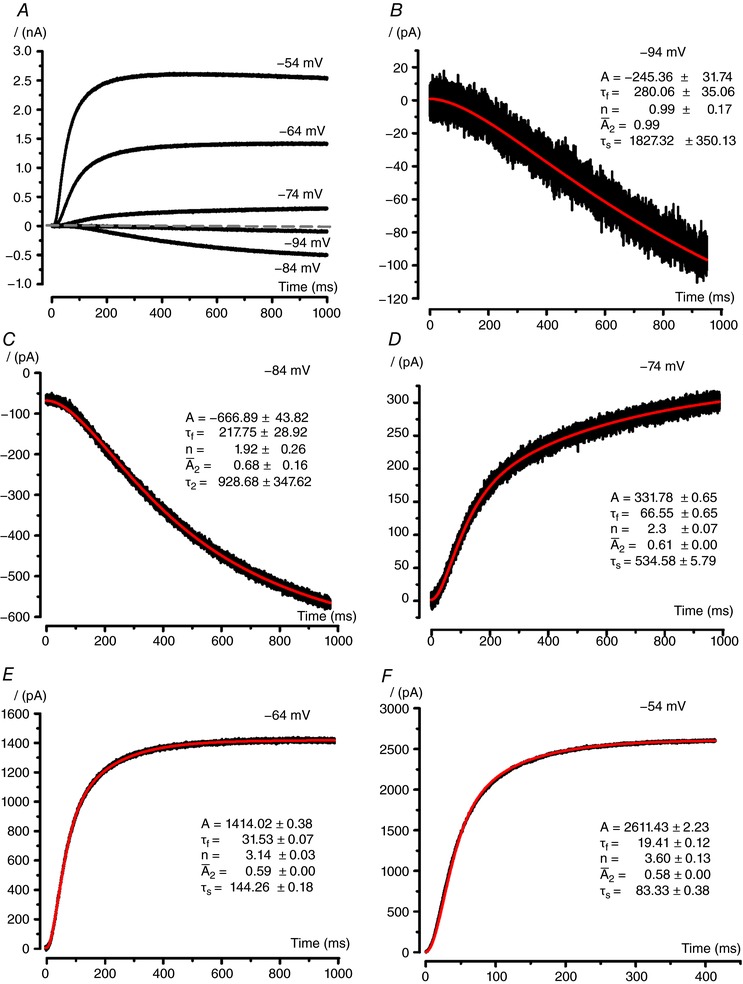
*I*_K,L_ kinetics *A*, representative whole‐cell currents recorded from a dissociated P24 type I hair cell, at the voltages indicated close to each trace, from a holding potential of –134 mV. *B*–*F*, single traces (same as in *A*), showing superimposed fitting with eqn (2) (see text). Function parameter values are also shown ± SD. No SD is shown at –94 mV for ${\bar{A}}_{2}$ because it was fixed for better fitting. Parameter amplitude and time constant have the same unit as shown in the axes.

Figure 5 shows *I*_K,L_ activation time course at voltages between –94 mV and –54 mV from *V*_hold_ of –134 mV. *I*_K,L_ inversion occurred between –80 mV and –70 mV (Fig. 5 *A*), i.e. slightly positive to *V*_eq_K^+^ (–80 mV). This recording was chosen because it allowed a good fit of *I*_K,L_ activation time course at all voltages, although in several other cells *I*_K,L_ was not detectable at –94 mV (cf. Fig. 4 *E*). *I*_K,L_ activation kinetics was particularly slow at –94 mV and –84 mV and was characterized by an initial lag, followed by a relatively fast sigmoid time course and a slowly increasing component (Fig. 5 *B*–*F*). The contribution of the slow component decreased with depolarization. We found that *I*_K,L_ onset phase could be consistently best fitted by the following function:

(2) $$\begin{array}{ccc} I(t) & = & A[{\bar{A}}_{1}{\left(1-e^{-t/\tau_{f}}\right)}^{n}\\ & & +\;{\bar{A}}_{2}\left(1-e^{-t/\tau_{s}}\right){\left(1-e^{-t/\tau_{f}}\right)}^{n}]+C, \end{array}$$

where *A* is the overall, steady‐state absolute current amplitude, ${\bar{A}}_{1}$ is the normalized, relative amplitude of the first component ${\bar{A}}_{1}=A_{1}/\left(A_{1}+A_{2}\right)$, whereas ${\bar{A}}_{2}$ is the normalized, relative amplitude of the second component ${\bar{A}}_{2}=A_{2}/\left(A_{1}+A_{2}\right)$. τ_f_ and τ_s_ are a fast and a slow time constants, respectively, and *n* is an exponential coefficient able to confer a sigmoidal behaviour to the current. It is important to note that the second component of the function is not independent from the first one, since the product ${\bar{A}}_{2}\left(1-e^{-t/\tau_{s}}\right)$ is multiplied by ${\left(1-e^{-t/\tau_{f}}\right)}^{n}$. The second component of the equation introduces a slowing down of the activation time course of a fraction of the total current, consistent with *I*_K,L_ recordings. The above equation includes the possibility that a homogenous population of ion channels activates with kinetics defined by two time constants.

The fitting function illustrated by eqn (2) was able to reproduce the *I*_K,L_ onset phase at all the *V*_test_ levels experimentally explored (Fig. 5 *B*–*F*). The exponential coefficient, *n*, was different at the various *V*_test_ levels, and its value was the lowest at the most negative *V*_test_ steps (1 at –94 mV; ∼2 at –84 mV) and increased for more depolarized *V*_test_ steps (∼3 at –64 mV, ∼4 at –44 mV – the fit at –44 mV is not shown). Analogous results were found in all dissociated type I hair cells and the obtained mean values are shown in Table 1. Given that the slower component of the current was most evident at hyperpolarized potentials, when multiple closed states are expected (Cole‐Moore shift) and that *n* increased with depolarization, the second component (${\bar{A}}_{2}$) can be regarded as the consequence of *I*_K,L_ channels transitioning to the open state(s) from intermediate (*n* ≤ 4) closed states. The number of closed states transited is in direct relation to *n*. By contrast, the first component of *I*_K,L_ onset can be regarded as the consequence of *I*_K,L_ channel transition to an open state, which is accessible at the end of the closed states chain. In other words, at relatively low depolarization levels (e.g. –90 to –80 mV), *I*_K,L_ channels have little chance of proceeding far through the sequence of closed states, and will more easily transit to open state(s) communicating with closed states located upstream in the activation chain.

**Table 1:** Fitting parameters for recorded and simulated *I*_K,L_

*V*_cond_	Parameter	Real current	Model current
−94 mV	τ_f_ (ms)	252 ± 70 (*n* = 10)	314
	*n*	1.05 ± 0.21 (*n* = 10)	1.28
	Ā_2_	0.90 ± 0.08 (*n* = 10)	0.99
	τ_s_ (ms)	1682 ± 693 (*n* = 10)	1872
−84 mV	τ_f_ (ms)	150 ± 50 (*n* = 12)	260
	*n*	1.98 ± 0.24 (*n* = 12)	2.25
	Ā_2_	0.71 ± 0.08 (*n* = 12)	0.89
	τ_s_ (ms)	666 ± 367 (*n* = 12)	946
−74 mV	τ_f_ (ms)	62 ± 24 (*n* = 9)	139
	*n*	2.42 ± 0.17 (*n* = 9)	2.76
	Ā_2_	0.64 ± 0.08 (*n* = 9)	0.74
	τ_s_ (ms)	417 ± 228 (*n* = 9)	276
−64 mV	τ_f_ (ms)	30 ± 4 (*n* = 12)	68
	*n*	3.05 ± 0.16 (*n* = 12)	3.34
	Ā_2_	0.61 ± 0.04 (*n* = 12)	0.62
	τ_s_ (ms)	131 ± 32 (*n* = 12)	105
−54 mV	τ_f_ (ms)	16 ± 3 (*n* = 5)	33
	*n*	3.64 ± 0.09 (*n* = 5)	3.71
	Ā_2_	0.59 ± 0.02 (*n* = 5)	0.56
	τ_s_ (ms)	77 ± 11 (*n* = 5)	53

**Figure 6:**
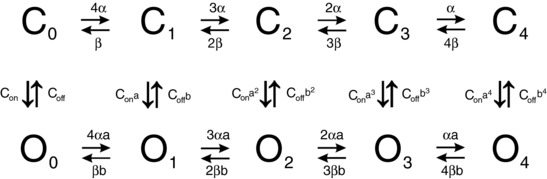
*I*_K,L_ channel gating scheme C_0_–C_4_: closed states; O_0_–O_4_: open states. All rate constants for vertical reactions are voltage independent; all rate constants for horizontal reactions are voltage dependent. The values of the kinetic parameters for the gating scheme were adjusted to fit the observed properties of *I*_K,L_, and were set as follows: α = α_0_exp(*V*/*k*_α_); β = β_0_exp(–*V*/*k*_β_), with α_0_ = 0.99 ms^−1^, β_0_ = 1.57·10^−6^ ms^−1^, *k*_α_ = *k*_β_ = 12.67 mV; *a* = 4.25; *b* = 0.38; C_on_ = 0.00045 ms^−1^; C_off_ = 0.8 ms^−1^.

**Figure 7:**
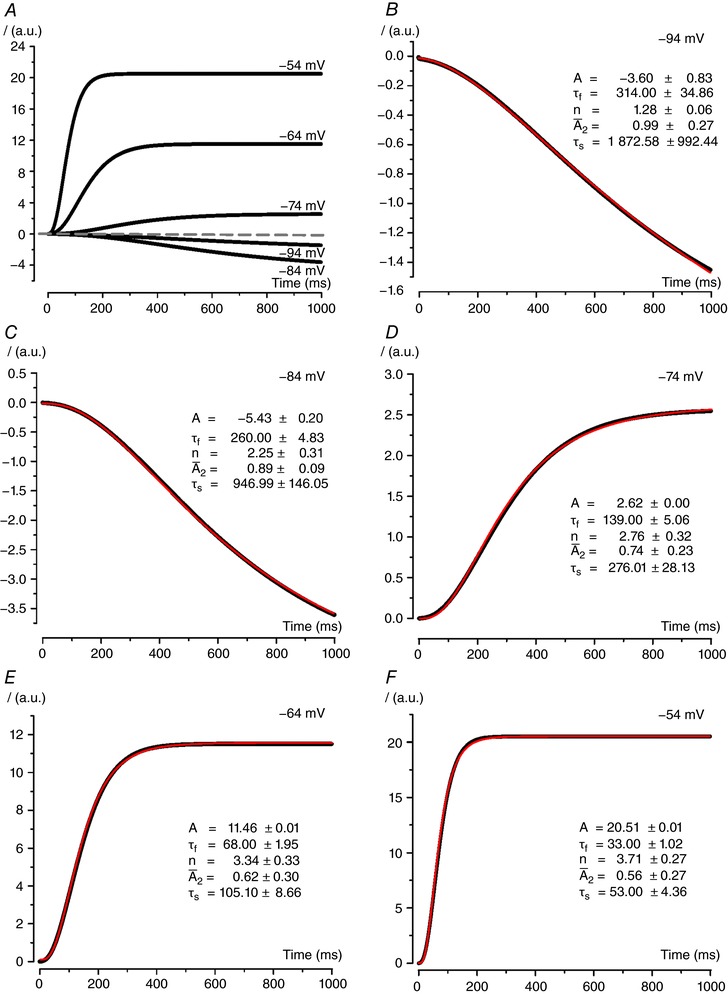
Activation kinetics of *I*_K,L_ generated by the gating model Amplitude values for modelled *I*_K,L_ (*A*–*F*) are shown in arbitrary units (a.u.). Fits with eqn (2) (red lines) are shown superimposed to generated traces. Function parameter values are also shown ± SD. Parameter amplitude and time constant have the same unit as shown in the axes.

The table shows the comparison between the mean values of fitting parameters obtained by applying eqn (2) as the fitting function for describing the time course of the onset phase of real *I*_K,L_ tracings (e.g. Fig. 5) and those obtained by applying eqn (2) as the fitting function for describing the time course of the onset phase of reconstructed tracings (Fig. 7) returned by the implementation of the kinetic model illustrated by the kinetic scheme reported in Fig. 6. Mean values (± SD) are from dissociated adult type I hair cells. Note that the number of cells investigated (*n*) is not the same at all potentials tested; this is due to the fact that at –94 mV (*n* = 10) *I*_K,L_ was sometimes not detectable, at –74 mV (*n* = 9) *I*_K,L_ was sometimes too small (close to its reversal potential) and at –54 mV (*n* = 5) a large *I*_K,L_ often produced a $V_{R_{s}}$ >6 mV. Membrane voltages were not corrected for the voltage drop produced by *I*_K,L_ across the residual *R*_s_.

Based on the above considerations, we devised the Markov kinetic scheme for the *I*_K,L_ channel illustrated in Fig. 6 (see Fig. 6 legend for kinetic parameters). In this model the transitions between closed states (C_0_ to C_4_) are voltage dependent, and at the end of the closed‐state chain is the open state O_4_ which is reached more easily and promptly in response to strong depolarization, thus increasing the weight of *I*_K,L_ ‘first’ onset component (τ_f_). Note that the transition to the final open state (O_4_) from the last closed state (C_4_), which represents the passage from the ‘activated’ state to the open state, occurs in a voltage‐independent manner and it is kinetically unrelated to the other transition rates. The closed states C_0_–C_3_ also interact, in a voltage‐independent manner, with ‘early’ open states O_0_–O_3_, transitions to which are favoured by slow or incomplete channel progression through the closed‐state chain (and hence, at low depolarization levels). The transitions through the chain of early open states will also be voltage dependent.

As shown in Fig. 7, *I*_K,L_ activation kinetics generated by the allosteric gating model faithfully reproduce those obtained by the experimental recordings (Fig. 5). Also, simulated *I*_K,L_ was consistently well fitted by eqn (2) (Fig. 7 *B*–*F*). The similarity between the parameters obtained by fitting the experimental currents and the model‐generated ones are shown in Table 1.

**Figure 8:**
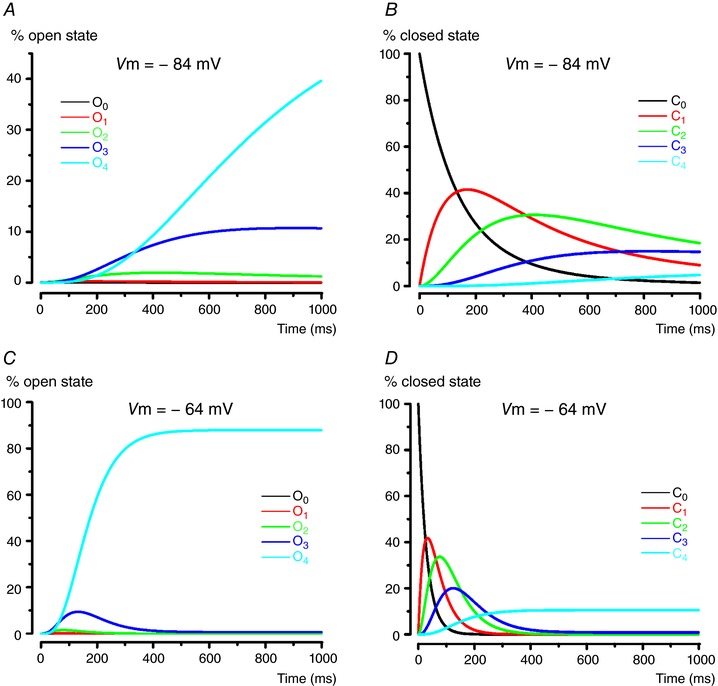
Open and closed states of modeled *I*_K,L_ activation at two representative potentials Percentage of *I*_K,L_ channels in the different open and closed states obtained with the model during a voltage step from –104 mV to –84 mV (*A* and *B*) and from –104 mV to –64 mV (*C* and *D*), respectively. Note that the importance of early open states decreases for stronger depolarization.

Figure 8 shows how the probabilities of distinct open and closed states change during *I*_K,L_ activation at two different voltages. Note that the probability of transiting through early open states decreases with depolarization. At –84 mV *I*_K,L_ channels have not reached a steady‐state condition in 1 s, when at –64 mV 90% of *I*_K,L_ channels are in O_4_ and the remaining 10% are mostly in C_4_.

**Figure 9:**
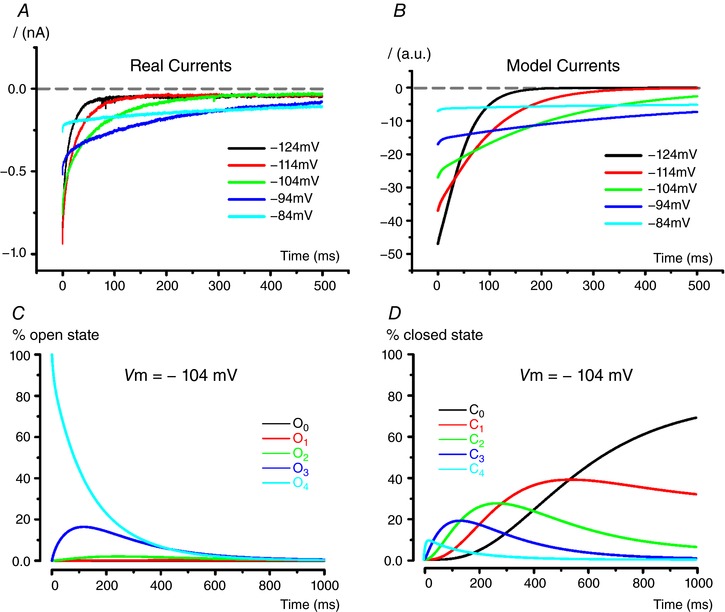
Deactivation kinetics of *I*_K,L_ *A*, experimental current traces recorded from a P29 dissociated type I hair cell. *B*, modelled current traces, in arbitrary units (a.u.). *C* and *D*, percentage of *I*_K,L_ channels in the different open and closed states, respectively, during a voltage step from –54 mV to –104 mV. Note that several open states are visited before all channels close.

The model also reproduced the deactivation time course of *I*_K,L_ (Fig. 9 *A* and *B*). The non‐mono‐exponential decay is consistent with *I*_K,L_ channels transiting through early open states during repolarization, as can be inferred by Fig. 9 *C* and *D*, which show the probabilities of distinct open and closed states during *I*_K,L_ full deactivation, generated by the same gating model reproducing *I*_K,L_ activation kinetics. The transition from O_4_ to C_4_ is presumably responsible for the initial, most rapid phase of deactivation.

## Discussion

In the present study we have shown that the residual calyx attached to the hair cell basolateral membrane alters the biophysical properties of *I*_K,L_ and as such it is likely to contribute to the previously reported variability in *I*_K,L_ properties. When the calyx was absent or largely reduced, *I*_K,L_ from mature type I hair cells exhibited complex activation and deactivation kinetics and steady‐state properties (half‐activation: –79.65 mV; steep voltage dependence: 2.84 mV), suggesting a homogeneous ion channels population. The native biophysical properties of *I*_K,L_ could be faithfully reproduced by a model of single‐channel elementary properties where the open state can be reached from five intercommunicating closed states. This model also explains the ability of *I*_K,L_ to change its activation kinetics at different membrane voltages, which could easily be confused for a combination of different K^+^ channel populations.

### *I*_K,L_ and the residual calyx

Similar to previous studies (Lim *et al*. 2011; Contini *et al*. 2012), we showed that *I*_K,L_ activation can produce K^+^ accumulation in the synaptic cleft between the basolateral membrane of the type I hair cell and the residual calyx. In addition, we have found that the inward *I*_K,L_, which is elicited by hyperpolarizing membrane potentials, can lower intercellular [K^+^]. The molecular architecture of the type I hair cell–calyx synapse has recently been partially elucidated. The protein Caspr is abundantly expressed at the postsynaptic membrane of the calyceal synapse (Sousa *et al*. 2009). Caspr is a transmembrane glycoprotein that contains an extracellular domain rich in laminin G‐like domains and a cytoplasmic domain that binds to the cytoskeleton. In the paranodal region of myelinated axons, Caspr binds to contactin to form a septate‐like junction which provides structural intercellular junctions between the axon and the glial cell (Faivre‐Sarrailh & Devaux, 2013). The expression of contactin has also been shown at the calyx inner membrane of the vestibular synapses (Lysakowski *et al*. 2011). The septate‐like junction represents a barrier for K^+^ diffusion out of the interstitial compartment between the axon and the glia (Salzer, 2003). It is possible that a similar ‘septate‐like’ junction is also present at type I hair cell synapses, thus functionally trapping K^+^ in the calyx synaptic cleft and as such depolarizing the calyx (Songer & Eatock, 2013).

### *I*_K,L_ ‘native’ properties

We found that the very slow activation and deactivation kinetics of *I*_K,L_ differed from those previously reported in mouse and rat utricle type I cells (Rüsch & Eatock, 1996; Wong *et al*. 2004). The difference was most obvious at voltages overlapping *I*_K,L_ activation range, where τ_d_ could be as slow as ∼1 s at –80 mV. By comparison Rüsch & Eatock (1996) reported a value of ∼20 ms at the same membrane voltage. This variability might be related to the different animal age investigated (P1–17 *vs*. ≥P18 here), although a residual calyx, which produces an apparent acceleration of *I*_K,L_ deactivation kinetics (Fig. 2 *A*), probably contributed. The residual calyx also caused a relaxation of outward *I*_K,L_ because the concomitant intercellular K^+^ accumulation produced a rightward shift of *V*_rev_K^+^, which will be more pronounced for larger outward K^+^ currents. This phenomenon may prevent identification of the slowest component of *I*_K,L_ activation kinetics (black arrows in Fig. 4 *B*). Our study also shows that all or at least most of the apparent inactivation of *I*_K,L_ (Figs 2 *A* and 3 *A*) was due to intercellular K^+^ accumulation. When the latter phenomenon was minimized, the slower component (τ_s_) of *I*_K,L_ could be recognized (black arrows in Fig. 4 *B*), and *I*_K,v_ could be distinguished from *I*_K,L_ (arrow in Fig. 4 *A*).

In the absence of the calyx, the steady‐state activation properties of *I*_K,L_ in adult (≥P18) type I hair cells were consistent among recordings (Fig. 4 *E*), with an average half‐activation voltage (*V*_1/2_) of –79.65 mV (± 4.35) and a slope factor of 2.84 mV (± 0.41). The small slope factor and similar *I*_K,L_ kinetics among cells indicate the presence of a homogenous population of ion channels. The above values are generally consistent with those previously reported for enzymatically dissociated rat vestibular type I hair cells (∼–75 mV; Chen & Eatock, 2000; Hurley *et al*. 2006), although these measurements varied largely among single cells (between –50 and –91 mV). The wide age range investigated (P10–45, Chen & Eatock, 2000; P2–26, Hurley *et al*. 2006) probably contributed to the larger variability compared to our results. Indeed, the slope of *I*_K,L_ activation curve narrowed after P18 (Hurley *et al*. 2006). However, the persistence of different amounts of residual calyx would also produce a significant variability in *I*_K,L_ activation properties. Of note, very long (several seconds) *V*_cond_ steps have to be used to reach a steady‐state level of *I*_K,L_ activation, which will significantly alter intercellular K^+^ concentration even in the presence of a small patch of residual calyx.

Finally, the *I*_K,L_ activation curve has been reported to shift rightward with time during ruptured, but not perforated, patch whole‐cell recording, suggesting that *I*_K,L_ was modulated by intracellular diffusible factors (Hurley *et al*. 2006). In contrast, we observed a leftward shift of *I*_K,L_ activation curve over time, explainable by the gradual deterioration of the calyx (Fig. 2 *B*). However, the study by Hurley *et al*. (2006) included rats as young as P2. Moreover, type I hair cells in the mouse seem to acquire *I*_K,L_ about a week earlier than rats, at least in the utricle (Géléoc *et al*. 2004). Therefore, it is possible that *I*_K,L_ ‘modulatability’ by intracellular factors is most critical during early neonatal development. The small variability in *I*_K,L_ *V*_1/2_ activation found in mature type I hair cells could be due to intracellular modulation (Hurley *et al*. 2006).

### A gating model for *I*_K,L_ channel

Fitting the activation and deactivation time course of *I*_K,L_ recorded from type I cells deprived of the calyx has allowed us to generate a model for the gating of the channel underlying *I*_K,L_, which faithfully reproduces the experimental data. The model was optimized to reproduce experimental protocols designed to isolate the essential channel features. The exponential raised to a variable power (${\bar{A}}_{1}$) in eqn (2) describes the variable sigmoidal activation and lag of *I*_K,L_ as a function of the voltage. These features are reproduced in the model with multiple closed‐state transitions before opening. This allows for minimal initial current followed by a steep rise, as observed experimentally (Figs 4 *B* and 5). In response to a small depolarization from a hyperpolarized membrane potential (e.g. from –134 mV to –94 mV), the power of the exponential is ∼ 1 (Fig. 5 *B* and Table 1), which in the model is described by the channel mainly opening from C_1_ to O_1_. Two time constants of activation (fast followed by slow) are evident, with a slow rise in current observed even for 1 s depolarization (Figs 4 *B* and 5). The slower activation component of *I*_K,L_ is described in eqn (2) by the mono‐exponential function multiplied by the same exponential raised to a power (${\bar{A}}_{2}$). In the model it is reproduced with transitions to the open state from different closed states, consistent with an allosteric voltage‐dependent gating of the channel. Finally, the complex deactivation kinetics observed experimentally (Figs 4 *D* and 8 *A*) are reproduced by the two closing pathways (Fig. 6).

Given the voltage dependency of the rate constants of *I*_K,L_, the channel will be mostly open at around the resting membrane potential (presumably ∼–70 mV), reacting slowly to membrane voltage changes. In other words, the channel appears to be designed to provide a large leak‐like K^+^ current in the whole range of the receptor potential. This leakage K^+^ current is substantially provided by the channel opening, even though not all of the voltage‐dependent sensors of the *I*_K,L_ channel have moved (i.e. through the early open states). This characteristic of *I*_K,L_ represents the major peculiarity in the model. In the HH classical gating model of the outward rectifier K^+^ channel, four voltage sensors (‘voltage‐sensing particles’) must move in order for the channel to open. This hypothesis was later supported by the discovery that the pore of most voltage‐dependent K^+^ channels has four structurally independent voltage sensor domains (Long *et al*. 2005). A kinetic scheme analogous to that used to model the properties of *I*_K,L_, where the K^+^ channel allows the flow of K^+^ following the movement of a variable number of voltage sensors (from 0 to 4), has recently been reported for the α‐subunit of the KCNQ1 (K_v_7.1) channel (Osteen *et al*. 2012). In this study, Osteen and colleagues derived the gating scheme by voltage‐clamp fluorometry, a technique that enables monitoring of voltage sensor(s) movement during ion flux through the channel. KCNQ1 is the only member of the K_v_ channel family so far described which gates in the same way as the channels underlying *I*_K,L_. Therefore, it is tempting to speculate that *I*_K,L_ is carried through KCNQ1 channels. However, in the peripheral vestibular system KCNQ1 channel subunits seem to be expressed by supporting dark cells but not by hair cells (Casimiro *et al*. 2001; Nicolas *et al*. 2001). Linopirdine and XE991, which are specific inhibitors of all KCNQ (1–5) channel isoforms, blocked a substantial fraction of the outward K^+^ current in neonatal, but not in adult type I hair cells (Hurley *et al*. 2006; Mann *et al*. 2013), consistent with the absence of KCNQ (4 and 5) channel subunits in adult mouse type I hair cells (Spitzmaul *et al*. 2013). In mice older than P16 the ether‐a‐go‐go‐related (erg, K_v_11) channel blockers E‐4031 or WAY‐123 were more effective in reducing the K^+^ current in adult type I hair cells (Hurley *et al*. 2006). However, erg channels show a c‐type inactivation that is not present in *I*_K,L_ (Hoshi & Armstrong, 2013). Thus, despite the presence of an extensive literature (for a recent review, see Meredith & Rennie, 2016), the full identity of the *I*_K,L_ channel remains unclear. By revealing the complex gating mechanism of the *I*_K,L_ channel the present study adds a key biophysical characterization step towards deciphering the nature of this elusive channel.

## Additional information

### Competing interests

None declared.

### Author contributions

Conception and design of the experiments: P.S., E.T., J.M. and S.M.; collection, analysis and interpretation of data: P.S., E.T., M.M., V.M., G.R., I.P., W.M., J.M. and S.M.; drafting the article or revising it critically for important intellectual content: P.S., E.T., M.M., V.M., G.R., I.P., W.M., J.M. and S.M. All authors approved the final version of the manuscript. Electrophysiological experiments were performed at the University of Pavia, Pavia, Italy.

### Funding

This work was supported by grants from the Ministero dell'Istruzione, dell'Università e della Ricerca to S.M. (Ref. 2009A9XJ7S) and the Wellcome Trust to W.M. (Ref. 102892).
